# Complete mitochondrial genome of the important bio-control fungus *Purpureocillium lilacinum* (Ophiocordycipitaceae, Hypocreales) and its phylogenetic analysis

**DOI:** 10.1080/23802359.2019.1699466

**Published:** 2019-12-13

**Authors:** Jianping Li, Guodong Zhang, Hong Yu, Luodong Huang, Wenbo Zeng, Yuanbing Wang

**Affiliations:** a Sanqi College, Wenshan University, Wenshan, China;; b Yunnan Herbal Laboratory, School of Life Sciences, Yunnan University, Kunming, China;; c The International Joint Research Center for Sustainable Utilization of Cordyceps Bioresources in China and Southeast Asia, Yunnan University, Kunming, China;; d The Research Center of Cordyceps Development and Utilization of Kunming, Yunnan Herbal Biotech Co. Ltd, Kunming, China;; e College of Life Science and Tecnology, Guangxi University, Nanning, China;; f Wenshan Biological Resources Development and Research Center, Wenshan University, Wenshan, China

**Keywords:** Mitochondrial genome, phylogenetic analysis, *Purpureocillium lilacinum*

## Abstract

*Purpureocillium lilacinum* is widely used as commercialized bio-control agents for controlling plant parasitic nematodes, as well as other insects and plant pathogens. In this study, the complete mitogenome of *P. lilacinum* was determined using the next-generation sequencing technology. The mitogenome is a circular molecule of 23,495 bp containing 15 protein-coding genes (PCGs), 2 rRNA (rnl and rns) genes and 22 tRNA genes. The overall base composition is 35.5% A, 36.0% T, 12.9% C and 15.6% G, with a CG content of 28.5%. Phylogenetic analysis inferred from 14 concatenated PCGs of 47 taxa shows that *P. lilacinum* is clustered with the genus *Tolypocladium* of Ophiocordycipitaceae and forms a separate clade with strong statistical support. This study contributes to our understanding about sytematics and evolutionary biology of cordycipitoid fungi.

Plant parasitic nematodes cause great economic losses in total amount to $157 billion annually around the world (Abad et al. 2008)*. Purpureocillium lilacinum*, previously named as *Paecilomyces lilacinus*, belongs to the family Ophiocordycipitaceae (Hypocreales) that is one of the most widely used as bio-control agents to control plant nematodes. It is commonly isolated from soil, plant roots, nematodes and insects, and is also an opportunistic pathogen of immunodeficient humans and other vertebrates (Luangsa-ard et al. 2011; Xie et al. 2016; de Sequeira et al. 2017). Its whole genome and comparative genomic analyses, as well as phylogenetic relationships with other related pathogens have been widely investigated (Luangsa-ard et al. 2011; Prasad et al. 2015; Wang et al. 2016). However, little is known about its mitogenome. This study aims to report the complete mitogenome of *P. lilacinum* and reveal its phylogenetic position in the order Hypocreales.

*Purpureocillium lilacinum*, parasitic on the adults of Coleoptera, was collected from Wenshan city of Yunnan in southwestern China (23°35'40”N, 103°50'49”E, alt. 1521 m). Among the collections, the strain WS1608 isolated from the synnema of *P. lilacinum* associated with an adult of Coleoptera was deposited at Wenshan Biological Resources Development and Research Center, Wenshan University, China. Mycelia cultured on PDA at 25 °C for 15 days under dark conditions were prepared to extract total genomic DNA using DNeasy Plant Genomic DNA purification Mini Kit (QIAGEN). The whole-genome sequencing was conducted by Novogene Co., Ltd. (Beijing, China) on the Illumina sequencing platform (HiSeq-PE150). The high-throughput sequencing data were assembled using the software SPAdes v. 3.11.0 (Bankevich et al. 2012). The mitochondrial genome was annotated using MFannot tool and ARWEN web server, combined with artificial correction technology. The Organellar Genome DRAW tool was used to drew the mitogenomic circular map (Lohse et al. 2007).

The annotated mitogenome of *P. lilacinum* WS1608 was submitted to GenBank under accession No. MN 635609. Its annotated mitogenome is a closed loop and represents a typical mitogenome of Hypocreales fungi. The total length of this mitogenome is 23,495 bp containing 15 protein-coding genes (PCGs), 2 rRNA (rnl and rns) genes and 22 tRNA genes. The overall base composition is as follows: 35.5% A, 36.0% T, 12.9% C and 15.6% G, with a CG content of 28.5%.

To determine the phylogenetic position of *P. lilacinum*, mitogenomic sequences of 47 taxa were downloaded from NCBI. The concatenated 14 PCGs from 47 mitogenomes were aligned using MUSCLE (Edgar 2004). Phylogenetic analysis was performed using the Bayesian inference (BI) method with the software MrBayes v.3.1.2 (Ronquist and Huelsenbeck 2003). The BI analysis was run on MrBayes v.3.1.2 for 5 million generations using the GTR + G + I model. Our phylogenetic topological structure is consistent with the previous study that was inferred from both BI and maximum likelihood (ML) analyses based on 27 taxa of the order Hypocreales (Figure 1) (Li et al. 2019). *Purpureocillium lilacinum* is clustered with the genus *Tolypocladium* of Ophiocordycipitaceae and forms a separate clade with strong statistical support by the posterior probabilities (BI-PP = 100%).

**Figure 1.**
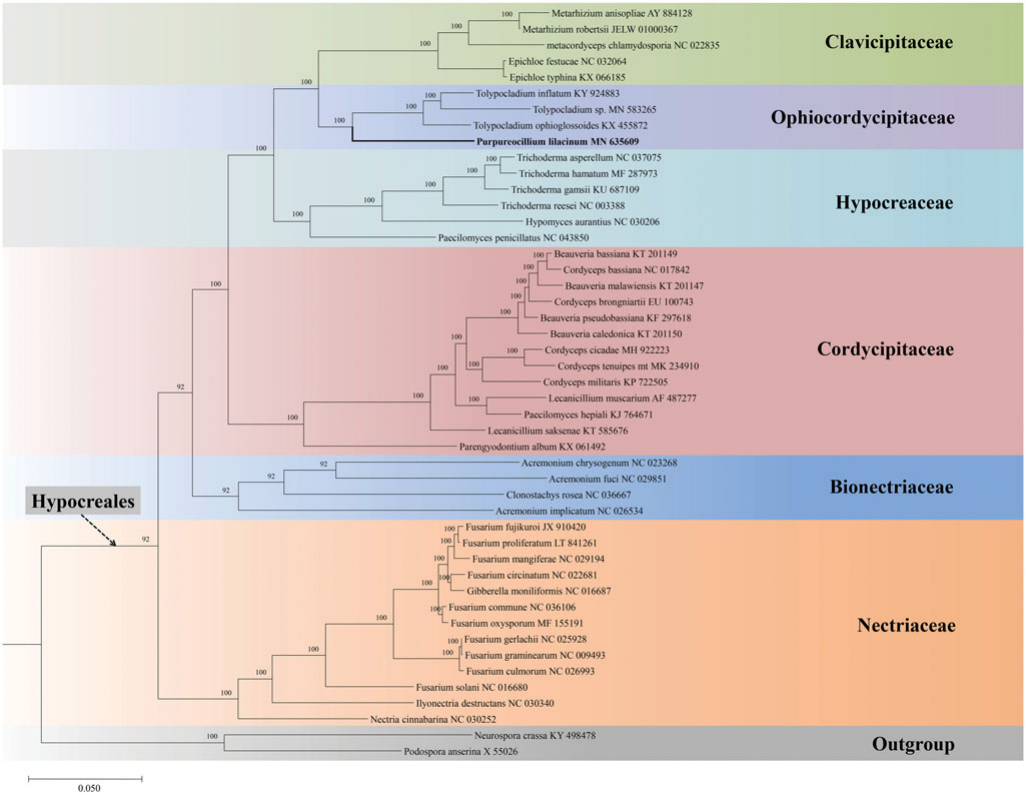
Phylogenetic analysis of 47 taxa in Sordariomycetes based on 14 concatenated mitochondrial protein-coding genes (PCGs). The 14 PCGs include subunits of the respiratory chain complexes (*cob, cox1, cox2, cox3*), ATPase subunits (*atp6, atp8, atp9*), NADH: quinone reductase subunits (*nad1, nad2, nad3, nad4, nad4L, nad5, nad6*). The phylogenetic tree is built by Bayesian inference (BI) and posterior probabilities are shown above internodes.
